# Single-cell RNA sequencing reveals a pro-fibrotic epithelial subpopulation contributing to endometrial dysfunction in polycystic ovary syndrome

**DOI:** 10.3389/fendo.2026.1873463

**Published:** 2026-09-03

**Authors:** Qiaoling Wang, Yunqing Zhi, Huiliang Xie, Jingwen Lang, Wanli Yang, Ajuan Liang, Xiuxian Zhu, Yonglun Fu

**Affiliations:** 1 Center for Reproductive Medicine, Shanghai First Maternity and Infant Hospital, School of Medicine, Tongji University, Shanghai, China; 2 Shanghai Key Laboratory of Maternal Fetal Medicine, Shanghai Institute of Maternal-Fetal Medicine and Gynecologic Oncology, Shanghai First Maternity and Infant Hospital, School of Medicine, Tongji University, Shanghai, China; 3 Reproductive Medicine Center, Renji Hospital, Shanghai Jiao Tong University School of Medicine, Shanghai, China

**Keywords:** endometrial dysfunction, epithelial cells, fibrosis, polycystic ovary syndrome, sc-RNA seq

## Abstract

**Background:**

Endometrial dysfunction in women with polycystic ovary syndrome (PCOS) significantly contributes to adverse pregnancy outcomes, including recurrent implantation failure and high miscarriage rates. However, the specific roles of individual cell types in this dysfunction, particularly in non-obese and obese PCOS patients, remain unclear.

**Methods:**

This study utilized single-cell RNA sequencing to comprehensively examine cellular heterogeneity in the proliferative-phase endometrium of non-obese PCOS, obese PCOS, and healthy controls.

**Results:**

Notably, we identified a distinct subpopulation of endometrial epithelial cells (EC) exhibiting a predisposition for epithelial-mesenchymal transition, characterized by elevated expression of decorin (DCN) and extracellular matrix 1 (ECM1) (DCN^high^ECM1^high^ EC2). The proportion of these cells was significantly higher in PCOS patients, potentially disrupting normal epithelial function and reducing endometrial receptivity. Analysis of cell-cell communication revealed a notable decrease in anti-fibrotic bone morphogenetic protein (BMP) signaling in fibroblasts targeting DCN^high^ECM1^high^ EC2, coupled with a marked increase in pro-fibrotic transforming growth factor beta (TGFβ) signaling in immune cells targeting DCN^high^ECM1^high^ EC2. These changes were further enhanced in obese PCOS patients compared to non-obese PCOS patients.

**Conclusions:**

The imbalance between the BMP and TGFβ pathways likely drives the fibrotic transformation of endometrial epithelial cells in both non-obese and obese PCOS patients. Obese phenotype further exacerbates the fibrotic tendency, and compromises endometrial receptivity. Our findings highlight potential therapeutic targets for managing endometrial dysfunction in PCOS.

## Introduction

Polycystic ovary syndrome (PCOS) is a complex endocrine disorder characterized by clinical hyperandrogenism, ovulatory dysfunction, and polycystic ovarian morphology (1). Epidemiological studies suggest that PCOS affects approximately 11-13% of women of reproductive age globally, posing a significant public health challenge with substantial socioeconomic implications (2). The etiology of PCOS is complex and multifactorial, involving genetic predisposition, dysregulation of the hypothalamic-pituitary-ovarian axis, insulin resistance, and chronic inflammatory responses (3). Infertility in PCOS is primarily attributed to oligo-ovulation or anovulation in patients with ovulatory dysfunction, and treatment strategies are typically aimed at improving ovulatory function (4–6). Despite the effectiveness of ovulation induction therapies in increasing ovulation rates, issues such as low implantation rates, reduced pregnancy rates, and high miscarriage rates persist (7–9). Consequently, poor reproductive outcomes in PCOS may also involve other factors, such as endometrial dysfunction (7, 10). Endometrial dysfunction in PCOS patients can be driven by various factors, including menstrual irregularities, hyperandrogenism, insulin resistance, immune dysregulation, and metabolic disturbances (7). Global gene expression profiling has revealed that distinct endometrial cell patterns in PCOS are linked to conditions like endometriosis, increased inflammation, delayed decidualization, and even endometrial cancer (11–13).

Obesity, a chronic metabolic disorder marked by excessive fat accumulation, is a well-established risk factor for numerous comorbidities, including type 2 diabetes, cardiovascular disease, and reproductive dysfunction (14, 15). As a common feature of PCOS, obesity exacerbates the disorder’s pathophysiology by intensifying insulin resistance, exacerbating hyperandrogenism, and promoting inflammation, all of which impair reproductive function at the ovarian, endometrial, and systemic levels (15, 16). While research suggests that intrinsic endometrial dysfunction exists in both non-obese and obese PCOS patients (17, 18), critical gaps remain regarding the specific contributions of individual cell types (e.g., epithelial, stromal, immune cells) to endometrial dysfunction, as well as potential differences in endometrial cellular composition between the non-obese and obese PCOS phenotypes. Addressing these gaps could pave the way for precision therapeutic strategies targeting PCOS-related endometrial pathologies.

This study employed single-cell RNA sequencing (scRNA-seq) to comprehensively characterize the cellular heterogeneity and cell-cell communication networks in the proliferative-phase endometrium of three distinct groups: non-obese PCOS, obese PCOS, and healthy controls. This high-resolution molecular and cellular atlas provides a detailed delineation of the pathological differences between these populations.

## Materials and methods

### Study design and participants

This study was conducted at the Reproductive Center of Shanghai First Maternity and Infant Hospital from July 2024 to December 2024. The study was approved by the Research Ethics Committee of Shanghai First Maternity and Infant Hospital (ethics approval reference KS24436) and was conducted in accordance with the Declaration of Helsinki. Written informed consent was obtained from all participants. All participants were women aged 20 to 35 years. The BMI of the control group and the non-obese PCOS (PCOS_non ob) group ranged from 18.5 to 23.9 kg/m^2^, while the obese PCOS (PCOS_ob) group had a BMI ≥ 28 kg/m^2^. PCOS diagnosis was based on the revised 2023 Rotterdam criteria (6), which include chronic anovulation or oligomenorrhea, clinical or biochemical hyperandrogenism, and polycystic ovarian morphology on ultrasound. Oligomenorrhea was defined as a menstrual cycle lasting 35 days to 3 months, and amenorrhea as the absence of menstruation for more than 3 months. Polycystic ovarian morphology was diagnosed if either of the following ultrasound criteria was met: ≥ 12 antral follicles (2–9 mm in diameter) per ovary, or ovarian volume > 10 cm^3^.

Exclusion criteria included other androgen excess or ovulatory disorders, such as hyperprolactinemia, Cushing’s syndrome, congenital adrenal hyperplasia, androgen-secreting tumors, or clinically significant thyroid dysfunction. The participants had taken metformin, oral contraceptives, or ovulation induction agents within the three months prior to the study. Additionally, patients with genetic or chromosomal abnormalities, severe adenomyosis or endometriosis, uterine malformations, severe hydrosalpinx, or moderate to severe intrauterine adhesions were excluded. Exclusion of severe adenomyosis and endometriosis was based on transvaginal ultrasound (TVUS) performed by a single experienced sonographer. Adenomyosis was diagnosed according to the MUSA criteria (presence of asymmetric myometrial thickening, subendometrial cysts, echogenic islands, and/or palisading vessels). Severe adenomyosis was defined as diffuse or focal disease with myometrial invasion depth ≥50% or maximal wall thickness ≥12 mm, accompanied by moderate-to-severe clinical symptoms (dysmenorrhea, menorrhagia). Ovarian endometriomas were diagnosed when a cyst with diffuse low-level echogenicity (‘ground-glass’ appearance) was observed. Patients with known histologically confirmed moderate-to-severe endometriosis (rASRM stage III–IV) were also excluded. Stage III (Moderate) is defined by a score of 16–40 points, characterized by multiple deep infiltrating lesions, small endometriomas in one or both ovaries, and partial membranous adhesions. Stage IV (Severe) is defined by a score exceeding 40 points, characterized by multiple deep infiltrating lesions, large endometriomas in one or both ovaries, and extensive dense adhesions, which may sometimes involve adhesion between the rectum and the posterior uterine wall.

### Single-cell dissociation from endometrial samples

For scRNA-seq, fresh endometrial biopsies from the proliferative phase were collected during hysteroscopic examination of a spontaneous menstrual cycle from nine women (three per group). All participants underwent standardized evaluation at enrollment (cycle days 2–4): basal hormone profiling (FSH, LH, E_2_, P) and transvaginal ultrasound for antral follicle count and baseline endometrial status (<5 mm). On the collection day (cycle days 10–12, hysteroscopic surgery), we repeated these assessments. Both time points consistently confirmed a follicular/proliferative phase, as evidenced by: (1) early follicular-phase E_2_ levels, (2) progesterone <1.0 ng/mL (excluding luteal transformation), and (3) proliferative endometrial morphology by ultrasound. Samples were immediately placed in cold tissue preservation solution for single-cell dissociation. The samples were washed with cold 1640 medium (Corning) supplemented with 0.04% BSA, sectioned, and digested with 0.2% collagenase type II (Gibco, USA) for 30–60 minutes at 37 °C with constant agitation. After digestion, the samples were filtered through a 40 μm cell strainer (Falcon, USA) and centrifuged at 300 g for 5 minutes to collect cells. Red blood cell lysis solution (Miltenyi Biotec) was added to the cell suspensions and incubated for 10 minutes at 4 °C to remove residual erythrocytes. The cells were then resuspended in 100 μl of RPMI 1640 medium supplemented with 0.04% BSA. Cell concentration and viability were assessed using a Luna-FL automated cell counter (Logos Biosystems, Korea), and samples were considered qualified if the viable cell rate exceeded 90%.

### Single-cell sequencing

The concentration of the single-cell suspension was adjusted to 700–1200 cells/μl. Library preparation and loading were performed according to the 10 × Genomics Chromium Next GEM Single Cell 3' Reagent Kits v3.1 (Catalog No.: PN-1000268) protocol. The constructed libraries were then subjected to high-throughput sequencing on the Illumina Nova 6000 PE150 platform. The scRNA-seq data have been deposited in the NCBI Gene Expression Omnibus (GEO) database under the accession number GSE338479.

### scRNA-seq data preprocessing

Sequencing and bioinformatics analysis were performed by OE Biotech Co., Ltd. (Shanghai, China). The raw reads generated from high-throughput sequencing were in FASTQ format. These data were processed using the official 10x Genomics software Cell Ranger (version 9.0.1) for quality assessment and alignment to the human reference genome (GRCh38). Further QC was carried out using the Scanpy package (version 1.10.4). Low-quality cells were filtered based on key metrics, including nUMI, nGene, and percent.mito. Cells were retained if they met the following criteria: nGene > 200, nUMI > 1000, mitochondrial percentage < 5%, and erythrocyte gene percentage < 5%. Potential doublets were removed using DoubletDetection (version 4.3.0). After QC, data were normalized using the Normalize_total function from the Scanpy package.

The top 2000 HVGs were identified using Scanpy’s highly_Variable_Genes function. Principal component analysis (PCA) was performed for dimensionality reduction. The harmony_integrate function from the harmonypy package (version 0.0.10) was used to correct batch effects. Results were visualized in two-dimensional space using Uniform Manifold Approximation and Projection (UMAP) for nonlinear dimensionality reduction. The cell proportion difference between groups was analyzed by Kruskal-Wallis test followed by *post-hoc* comparisons. Marker genes were identified using the rank_genes_groups function from the Scanpy package, which involves finding genes that were differentially upregulated in each cell type compared to other populations. These genes served as potential markers for each cell type. Visualization of the identified marker genes was performed using the VlnPlot and FeaturePlot functions from the Seurat software package (version 4.0.0).

### DEG and enrichment analyses

DEG screening was performed using the rank_genes_groups function in Scanpy (method = “wilcoxon”). Significant DEGs were identified based on an adjusted P-value < 0.05 and |log_2_(fold change)| > 0.58. The Benjamini-Hochberg (BH) procedure was applied to control the false discovery rate (FDR) in both differential expression analysis and marker gene identification. GO and KEGG enrichment analyses were conducted for these DEGs using the hypergeometric distribution test.

GSVA enrichment analysis began by downloading and organizing background gene set files from the KEGG (https://www.kegg.jp/) and GO (https://geneontology.org/) databases using the GSEABase package (version 1.44.0). The GSVA package (version 1.30.0) was then used to calculate pathway activity scores for individual cells. The LIMMA package (version 3.38.3) was employed to evaluate differences in signaling pathway activities across different groups.

### Pseudo-time analysis

Cell differentiation trajectory inference was performed using the Monocle2 package (version 2.9.0). The process began by converting the Seurat object to a CellDataSet object using the importCDS function from Monocle2. Genes (q-val < 0.01) for cell ordering were selected using the differentialGeneTest function. Dimensionality reduction and clustering were then performed with the reduceDimension function. Finally, the differentiation trajectory was inferred using the orderCells function.

### Cell-cell communication analysis

The CellChat R package (version 2.1.2) was used to analyze ligand-receptor interactions between cells. Initially, the normalized expression matrix was imported, and a CellChat object was created using the createCellChat function. Preprocessing was performed with default parameters using the identifyOverExpressedGenes, identifyOverExpressedInteractions, and projectData functions. Potential ligand-receptor interactions were then calculated using the computeCommunProb, filterCommunication (min.cells = 10), and computeCommunProbPathway functions. Finally, intercellular communication networks were aggregated with the aggregateNet function.

### Immunofluorescence staining

Endometrial tissues were fixed in 4% paraformaldehyde (w/v), embedded in paraffin, and sectioned into 5 μm thick slices. Antigen retrieval was performed using 1 mM Tris-EDTA buffer (pH 8.0) at 100 °C for 15 minutes, followed by blocking with BSA for 30 minutes. The sections were incubated overnight at 4 °C with a mouse anti-EPCAM primary antibody (1:2000 dilution; GB12274, Servicebio, China) with a rabbit anti- ESRα primary antibody (1:800 dilution; GB111843, Servicebio, China), or with a rabbit anti-αSMA primary antibody (1:500 dilution; GB111364, Servicebio, China), or with a rabbit anti-p-SMAD2/3 primary antibody (1:500 dilution; GB111844, Servicebio, China). After washing, the sections were treated by a goat anti-mouse Alexa Fluor 488 secondary antibody (1:400, GB25301, Servicebio, China), and a goat anti-rabbit CY3 secondary antibody (1:300, GB21303, Servicebio, China) for 30 minutes at 37 °C. Coverslips were mounted using an antifade mounting medium with DAPI (G1012, Servicebio, China), and images were captured using an automated fluorescence microscopy system (Pannoramic scanner, 3DHISTECH, Budapest, Hungary) at the same exposure time. The median fluorescence intensity of ESRα staining, αSMA staining or p-SMAD2/3 staining co-expressed with EPCAM was quantified in 3 different views per slide from 3 endometrial samples in each group using Image J (NIH).

### Culture and treatment of cell lines

Ishikawa cells (human endometrial epithelial cell line) were provided by Procell (CL-0283, Wuhan, China), and were cultured in DMEM/F12 supplemented with 10% FBS and 1% PS. Cells were treated with 10 nM estradiol (E2) for 6 days to mimic the proliferative endometrium, and 100 nM dihydrotestosterone (DHT) was applied to induce a PCOS phenotype (19). Following treatment, cells were washed with PBS and harvested in lysis buffer for subsequent RNA extraction.

### RNA extraction and real-time PCR

Total RNA was extracted from cell lines or human endometrial tissues using the EastepTM Super Total RNA Extraction Kit (Promega Biotech Co., China) and reverse transcribed to cDNA using PrimeScript Reverse Transcript Master Mix (TaKaRa, Japan). Real-time quantitative PCR was performed using Applied Biosystems QuantStudioDx (Thermo Fisher Scientific, USA) with the universal SYBR Green qPCR Master Mix (Vazyme Biotech, China). The following primers were used: ACTB, forward CATGTACGTTGCTATCCAGGC, reverse CTCCTTAATGTCACGCACGAT; YWHAZ, forward CCTGCATGAAGTCTGTAACTGAG, reverse GACCTACGGGCTCCTACAACA; DCN, forward ATGAAGGCCACTATCATCCTCC, reverse GTCGCGGTCATCAGGAACTT; ECM1, forward GCTTCACGGCTACAGGACAG, reverse GAGGCTTCGGGATAGGGGT; BMPR1A, forward TGAAATCAGACTCCGACCAGA, reverse TGGCAAAGCAATGTCCATTAGTT; BMPR1B, forward CTTTTGCGAAGTGCAGGAAAAT, reverse TGTTGACTGAGTCTTCTGGACAA; TGFβ1, forward CAATTCCTGGCGATACCTCAG, reverse GCACAACTCCGGTGACATCAA; ACTA2, forward AAAAGACAGCTACGTGGGTGA, reverse GCCATGTTCTATCGGGTACTTC; VIM, forward AGTCCACTGAGTACCGGAGAC, reverse CATTTCACGCATCTGGCGTTC; SNAI1, forward TCGGAAGCCTAACTACAGCGA, reverse AGATGAGCATTGGCAGCGAG; ZEB1, forward GATGATGAATGCGAGTCAGATGC, reverse ACAGCAGTGTCTTGTTGTTGT. All the samples were run in duplicate in a 384-well reaction plate. The comparative ΔΔCT method was used to evaluate the relative mRNA levels of the housekeeping gene ACTB or YWHAZ.

### Statistical analysis

Continuous variables are presented according to their distribution: normally distributed data are expressed as mean ± SD, while non-normally distributed data are reported as median ± interquartile range (IQR). For statistical analysis, normally distributed data were compared using Student’s t-test or One-way ANOVA, and non-normally distributed data were analyzed using the two-tailed Mann–Whitney U test. All analyses were conducted using SPSS version 27.0. A p-value < 0.05 was considered statistically significant.

## Results

### Single-cell atlas of the endometrium in PCOS patients stratified by obesity

Endometrial biopsies from the functional layer were collected during the proliferative phase from nine women, divided into three groups: Control (age: 31.7 ± 1.5 years, body mass index [BMI]: 21.9 ± 1.3 kg/m^2^), PCOS_non-ob (age: 30.0 ± 2.0 years, BMI: 21.5 ± 1.9 kg/m^2^), and PCOS_ob (age: 29.3 ± 4.7 years, BMI: 29.3 ± 2.0 kg/m^2^). Compared to controls, serum LH and testosterone showed an increasing trend in the PCOS_non-ob group, whereas both were significantly elevated in the PCOS_ob group. AMH was significantly increased only in the PCOS_non-ob group. Both PCOS subgroups exhibited significantly elevated HOMA-IR compared to controls (non-obese PCOS: 2.41 ± 0.46, p<0.01; obese PCOS: 2.86 ± 1.01, p<0.05). Clinically, both PCOS groups presented with oligomenorrhea (cycle length 60–120 days in non-obese PCOS, 60–180 days in obese PCOS) and met the PCOM criteria (≥20 antral follicles per ovary). No patient in either group exhibited clinical hyperandrogenism, and none of the participants had taken metformin, oral contraceptives, or ovulation induction agents within the three months prior to the study. Direct comparison between the two PCOS subgroups revealed no significant differences except for BMI and fasting glucose levels, which were elevated in the PCOS_ob group (**Supplementary Table 1**).

**Figure 1:**
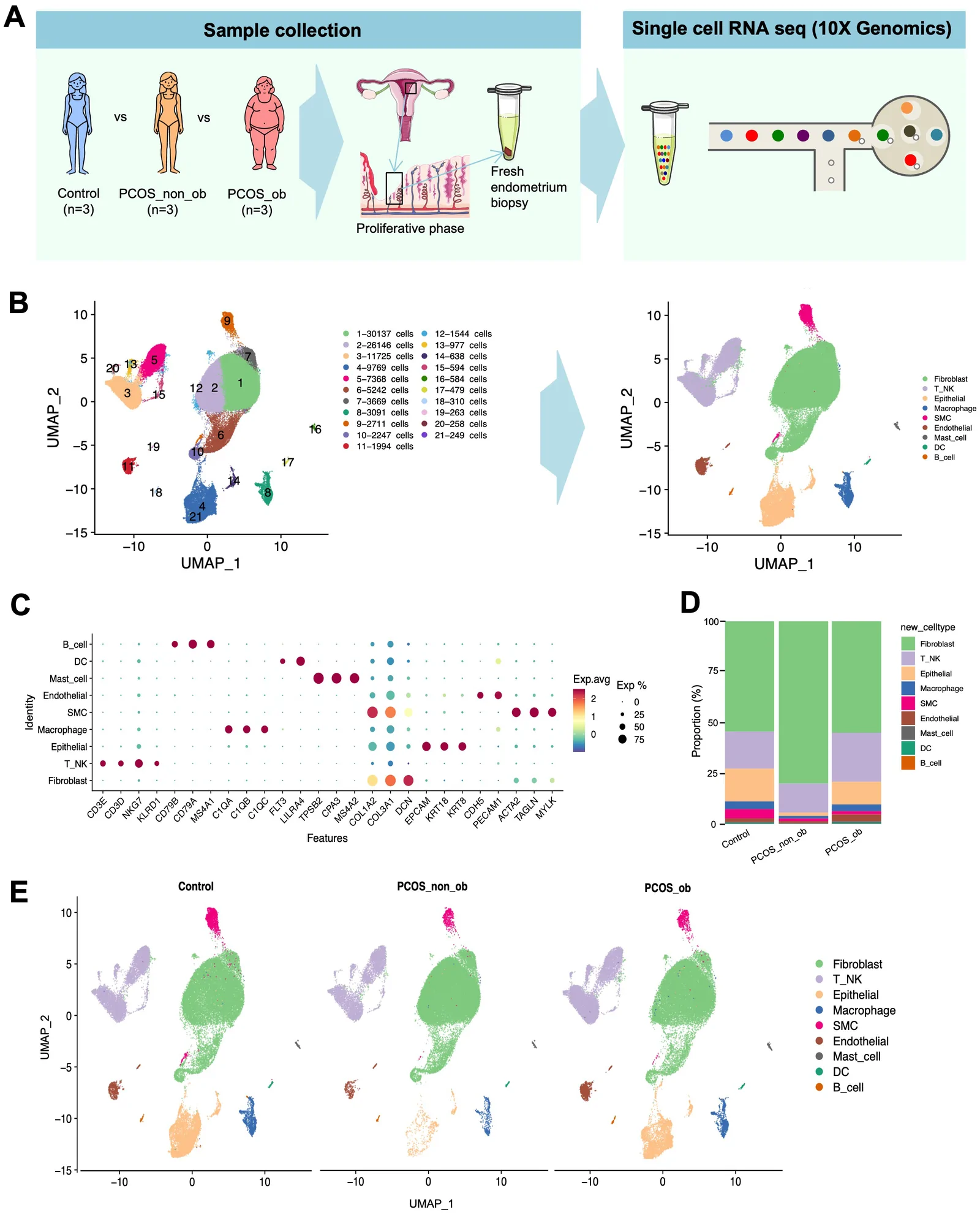
Single-cell profiling of the endometrium in non-obese PCOS, obese PCOS, and control groups. **(A)** Schematic overview of sample collection and scRNA-seq using 10x Genomics. **(B)** UMAP projections of integrated scRNA-seq data from 9 patients, revealing 9 distinct cell type-specific clusters. **(C)** Dot plots illustrating marker gene expression for each major cluster. **(D)** Bar plots depicting the proportion of each major cluster in the non-obese PCOS (PCOS_non ob), obese PCOS (PCOS_ob), and control groups. **(E)** UMAP showing the distribution of major clusters across the PCOS_non ob, PCOS_ob, and control groups.

Single-cell transcriptome libraries were generated using the droplet-based 10X Genomics Chromium System (**Figure 1A**). After computational quality control (QC) and transcriptome integration, 43,534 cells from the control group, 42,815 cells from the PCOS_non-ob group, and 46,401 cells from the PCOS_ob group were retained for further analysis. High-quality cells were integrated for graph-based clustering using UMAP for dimensional reduction, and major cell types were annotated based on cluster-specific marker genes. In total, the sequenced endometrial cells were classified into nine clusters: Stromal_fibroblasts (marker genes: COL1A2, COL3A1, DCN), T_Natural killer cells (T_NK, marker genes: CD3E, CD3D, NKG7, KLRD1), Epithelial cells (marker genes: EPCAM, KRT18, KRT8), Macrophages (marker genes: C1QA, C1QB, C1QC), Smooth muscle cells (SMC, marker genes: ACTA2, TAGLN, MYLK), Endothelial cells (marker genes: CDH5, PECAM1), Mast cells (marker genes: TPSB2, CPA3, MS4A2), Dendritic cells (DC, marker genes: FLT3, LILRA4), and B cells (marker genes: CD79B, CD79A, MS4A1) (**Figures 1B, C**). The dot plot and feature plot of all marker genes are shown in **Figure 1C** and **Supplementary Figure 1**. Notably, the total number and proportion of epithelial cells (ECs) were reduced in the PCOS_non-ob group (average cell number: 195, average proportion: 1.58%) and slightly decreased in the PCOS_ob group (average cell number: 1,423, average proportion: 10.48%) compared to the control group (average cell number: 1,933, average proportion: 15.88%), although these were not statistically significant due to the small sample size (**Figures 1D, E**, **Supplementary Figure 2**). No substantial changes in other cell types were observed across the groups.

### Subcluster of the main endometrial cell types in PCOS patients stratified by obesity

**Figure 2:**
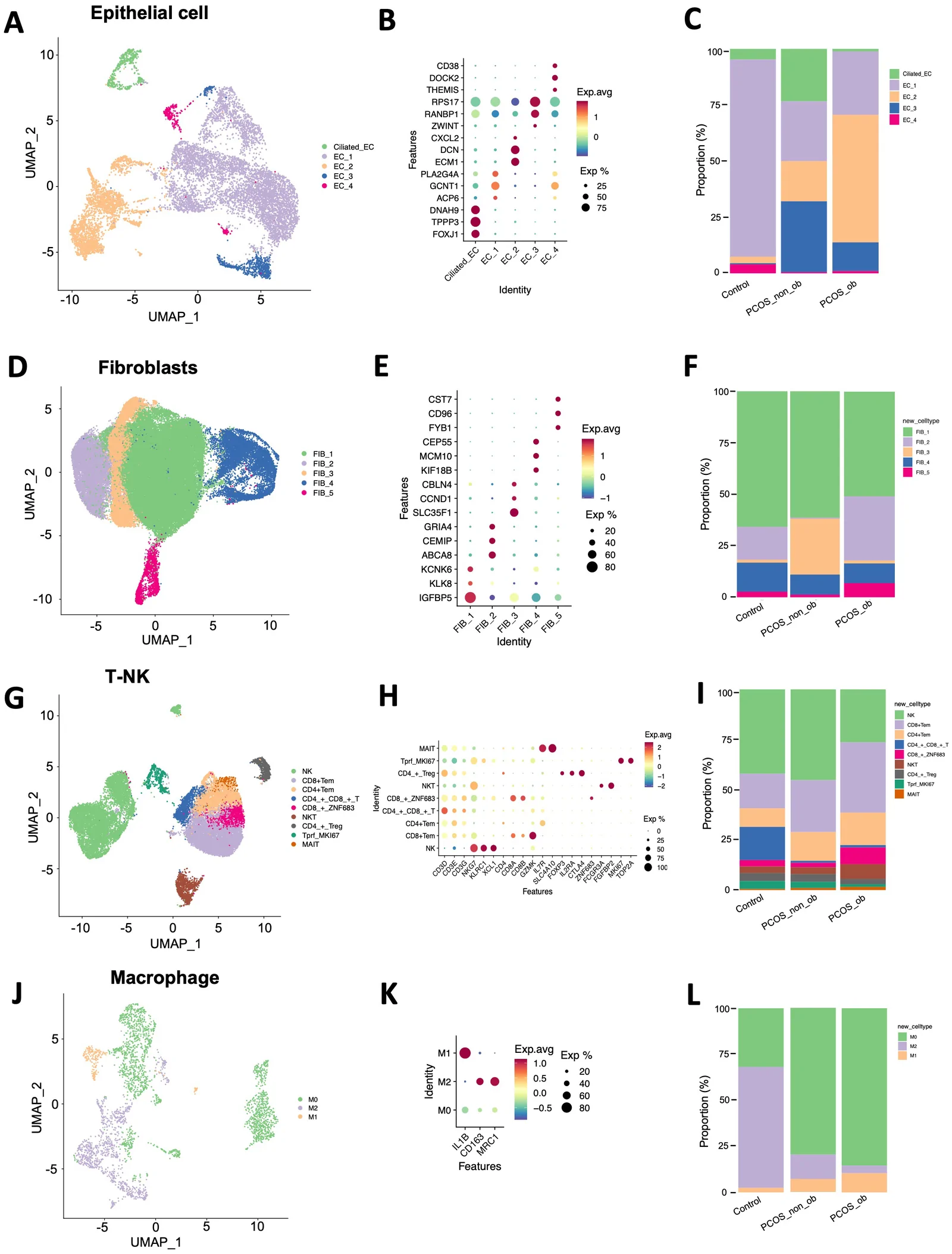
Subclustering of endometrial cell types in non-obese PCOS, obese PCOS, and control groups. **(A)** UMAP illustrating the subcluster distribution of epithelial cells (EC). **(B)** Dot plots showing the marker gene expression characterizing EC subclusters. **(C)** Bar plots representing the proportion of EC subclusters in the PCOS_non ob, PCOS_ob, and control groups. **(D)** UMAP depicting the subcluster distribution of fibroblasts (FIB). **(E)** Dot plots showing the marker gene expression characterizing FIB subclusters. **(F)** Bar plots displaying the proportion of FIB subclusters in the PCOS_non ob, PCOS_ob, and control groups. **(G)** UMAP demonstrating the subcluster distribution of T-NK cells. **(H)** Dot plots depicting the marker gene expression characterizing T-NK subclusters. **(I)** Bar plots illustrating the proportion of T-NK subclusters in the PCOS_non ob, PCOS_ob, and control groups. **(J)** UMAP showing the subcluster distribution of macrophages. **(K)** Dot plots representing the marker gene expression characterizing macrophage subclusters. **(L)** Bar plots displaying the proportion of macrophage subclusters in the PCOS_non ob, PCOS_ob, and control groups.

Subset analysis of endometrial ECs identified five distinct clusters: Ciliated EC, EC1, EC2, EC3, and EC4 (**Figure 2A**, **Supplementary Figure 3A**). The number of EC cells per sub-cluster per group were shown in **Supplementary Table 2**. Since the cell numbers of EC3 and EC4 were too low, these sub-clusters were no longer discussed. Gene Ontology (GO) enrichment analysis and Gene Set Variation Analysis (GSVA) of differentially expressed genes (DEGs) across these clusters revealed that ciliated ECs were enriched in processes related to axoneme assembly and cilium movement, with high expression of FOXJ1, TPPP3, and DNAN9 (**Figure 2B**, **Supplementary Figure 3C**). EC1 showed enrichment in glycerophospholipid biosynthesis and metabolic processes, with high expression of ACP6, GCNT1, and PLA2G4A, and was thus termed glycolipid metabolism-related EC1 (**Figure 2B**, **Supplementary Figure 3C**). Notably, for the first time, a subset of EC2 characterized by high expression of fibroblast-associated genes decorin (DCN) and extracellular matrix 1 (ECM1)—both linked to fibrosis and tissue repair—was identified (20–22) (**Figure 2B**, **Supplementary Figures 3B, C**). This subset was designated as DCN^high^ECM1^high^ EC2. EC1 represented the largest proportion of endometrial ECs, followed by DCN^high^ECM1^high^ EC2 (**Figures 2A, C**). Compared to the control group, the proportion of EC1 was significantly decreased in PCOS_non-ob group (P<0.001) and PCOS_ob group (P<0.05) compared to the control group. While the proportion of EC2 was significantly increased in PCOS_non-ob and PCOS_ob group (P<0.05) (**Figures 2A, C**, **Supplementary Figures 3D, E**). This suggests that epithelial-mesenchymal transition (EMT) may have occurred in DCN^high^ECM1^high^ EC2 in PCOS patients. The increased presence of DCN^high^ECM1^high^ EC2 may replace the functionally normal EC1, leading to impaired endometrial receptivity in PCOS. Further analysis will primarily focus on the characteristics of DCN^high^ECM1^high^ EC2.

The other three relatively abundant endometrial cell groups—fibroblasts (FIB), T-NK cells, and macrophages—were further divided into subclusters. FIB, the most abundant cell type in the endometrium, was subdivided into five clusters: FIB1, FIB2, FIB3, FIB4, and FIB5 (**Figure 2D**). GSVA functional analysis and marker gene profiling revealed heterogeneity among these subclusters (**Figure 2E**, **Supplementary Figure 4A**). Compared to the control group, the proportion of FIB cells was not significantly changed in the PCOS_non-ob group and the PCOS_ob group (**Figure 2F**). T-NK cells were classified into nine clusters: NK cells, CD8^+^ Tem cells, CD4^+^ Tem cells, CD4^+^CD8^+^ T cells, CD8^+^ ZNF683, NKT cells, CD4^+^ Treg cells, Tprf_MKI67, and MAIT cells (**Figure 2G**). Marker genes for these clusters are shown in **Figure 2H**. The proportion of subclusters of TNK cells was not significantly changed between groups (**Figure 2I**). Macrophages were classified into three subtypes: M0, M1 (marker gene: IL1B), and M2 (marker gene: CD163, MRC1) (**Figures 2J, K**). The proportion of subclusters of macrophages was not significantly changed between groups (**Figure 2L**).

### In-depth analysis of epithelial subclusters in PCOS patients stratified by obesity

**Figure 3:**
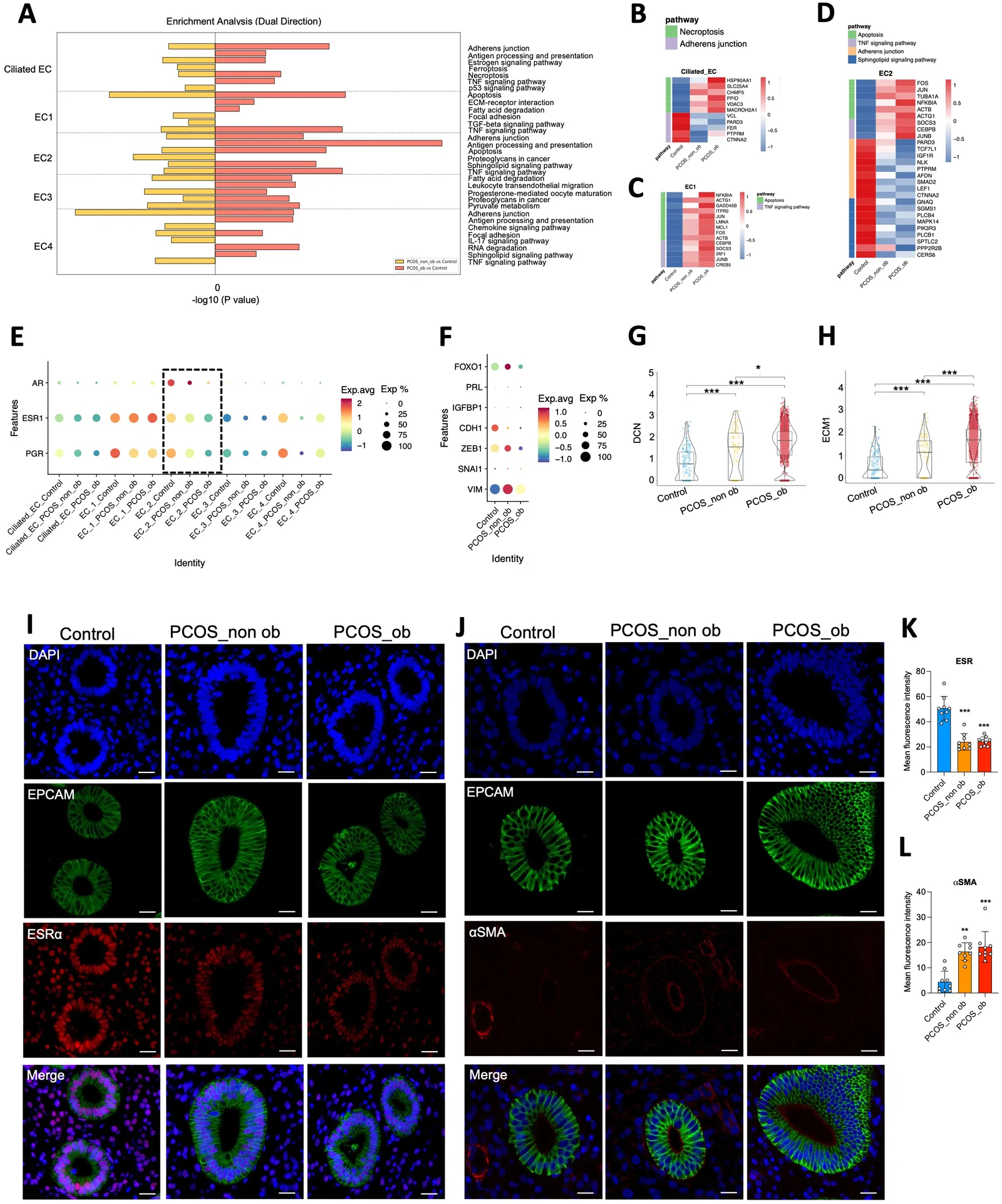
In-depth analysis of epithelial subclusters in non-obese PCOS, obese PCOS, and control groups. **(A)** KEGG enrichment analysis of DEGs in each epithelial subcluster, comparing the PCOS_non ob group *vs.* control group and PCOS_ob group *vs.* control group. **(B-D)** Heatmap showing DEGs in KEGG pathways in epithelial subclusters between the PCOS_non ob group, PCOS_ob group, and the control group. **(E)** Dot plots displaying the expression of androgen receptor (AR), estrogen-α receptor (ESR1), and progesterone receptor (PGR) in epithelial subclusters across the three groups. Dashed boxes highlight the expression of these three genes in EC2 across the groups. **(F)** Dot plots displaying the expression of EMT makers (VIM, SNAI1, ZEB1, CDH1) and decidualization markers (IGFBP1, PRL, FOXO1) in DCN^high^ECM1^high^ EC2 of three groups. **(G, H)** Violin plots illustrating the log-transformed expression of DCN and ECM1 in DCN^high^ECM1^high^ EC2 cells in the PCOS_non ob, PCOS_ob, and control groups. **(I, J)** Representative immunofluorescence staining for EPCAM (green) co-expressed with ESRα (Red), or αSMA (Red). Nuclei were stained with DAPI (blue). Scale bars, 25μm. **(K, L)** The mean fluorescence intensity of ESRα or αSMA co-expressed with EPCAM in the three groups. ^*^P < 0.05, ^**^P < 0.01, ^***^P < 0.001, vs. the control group.

Kyoto Encyclopedia of Genes and Genomes (KEGG) enrichment analysis of DEGs between the PCOS_non-ob versus control group and PCOS_ob versus control group is shown in **Figure 3A**. In ciliated EC cells, upregulated genes (e.g., HSP90AA1, SLC25A4, CHMP5) in both the PCOS_non-ob and PCOS_ob groups were significantly enriched in the necroptosis pathway, while downregulated genes (e.g., VCL, PARD3, FER) were notably enriched in adherens junction-related pathways (**Figure 3B**). In EC1 and DCN^high^ECM1^high^ EC2, genes upregulated by apoptosis and TNF signaling pathways, such as JUN, FOS, and NFKB1A, were enriched in PCOS_non-ob group and further elevated in PCOS_ob group (**Figures 3C, D**). These results imply that the endometrial microenvironment in the PCOS_ob group exhibits a more pronounced tendency toward inflammatory and apoptotic pathways compared with the PCOS_non-ob group. Conversely, the adherens junction and sphingolipid signaling pathways were enriched by downregulated genes (e.g., IGF1R, LEF1, MAPK14) in DCN^high^ECM1^high^ EC2 (**Figure 3D**). Estrogen receptor-α (ESR1), progesterone receptor (PGR), and androgen receptor (AR) play essential roles in regulating endometrial proliferation and differentiation (23). Expression of ESR1 was downregulated in ciliated EC and DCN^high^ECM1^high^ EC2 in both the PCOS_non-ob and PCOS_ob groups compared to the control group (**Figure 3E**, **Supplementary Figure 4B**). PGR expression was significantly decreased in ciliated EC, EC1 and DCN^high^ECM1^high^ EC2. AR expression was also significantly reduced in ciliated EC and DCN^high^ECM1^high^ EC2. Especially, the downregulation of these three genes was more pronounced in DCN^high^ECM1^high^ EC2 in the PCOS_ob group compared to the PCOS_non-ob group (**Figure 3E**, **Supplementary Figure 4B**). This suggests that the obese phenotype exacerbates endometrial impairment in PCOS patients. The average expression of EMT markers VIM and SNAI1 was obviously increased in the PCOS_non-ob group and PCOS_ob group compared to the control group. The average expression of ZEB1 was increased in the PCOS_non-ob group but decreased in the PCOS_ob group. The average expression of CDH1 was decreased in both the PCOS_non-ob group and the PCOS_ob group. In total, the expression of EMT markers including VIM, SNAI1, ZEB1 was increased in DCN^high^ECM1^high^ EC2 of PCOS groups (**Figure 3F**), suggesting that EMT is activated in endometrial epithelial cells of PCOS patients, conferring a mesenchymal-like phenotype. The decidualization markers IGFBP1 and PRL showed a very low expression, and FOXO1 expression was increased in PCOS_non-ob group (**Figure 3F**). The expression of DCN and ECM1 was significantly increased in DCN^high^ECM1^high^ EC2 in the PCOS_non-ob groups compared to the control group. This increasing was even more pronounced in PCOS_ob group compared to the PCOS_non-ob group (**Figures 3G, H**). Immunofluorescence staining revealed that, compared to controls, the PCOS groups exhibited a significant decrease in ESR protein co-expression with the epithelial marker EPCAM, alongside a marked increase in the epithelial expression of the classical fibrosis marker α-SMA (**Figures 3I–L**). It was not significantly changed between the PCOS_ob group and the PCOS_non-ob group. These results indicate that both the proportion and functionality of endometrial EC subtypes are dysregulated in PCOS patients. The increased proportion of DCN^high^ECM1^high^ EC2 and their aberrant functionality may be pivotal factors contributing to reduced endometrial receptivity and recurrent implantation failure observed in these individuals.

### Pseudotime reconstruction of epithelial cells in PCOS patients stratified by obesity

**Figure 4:**
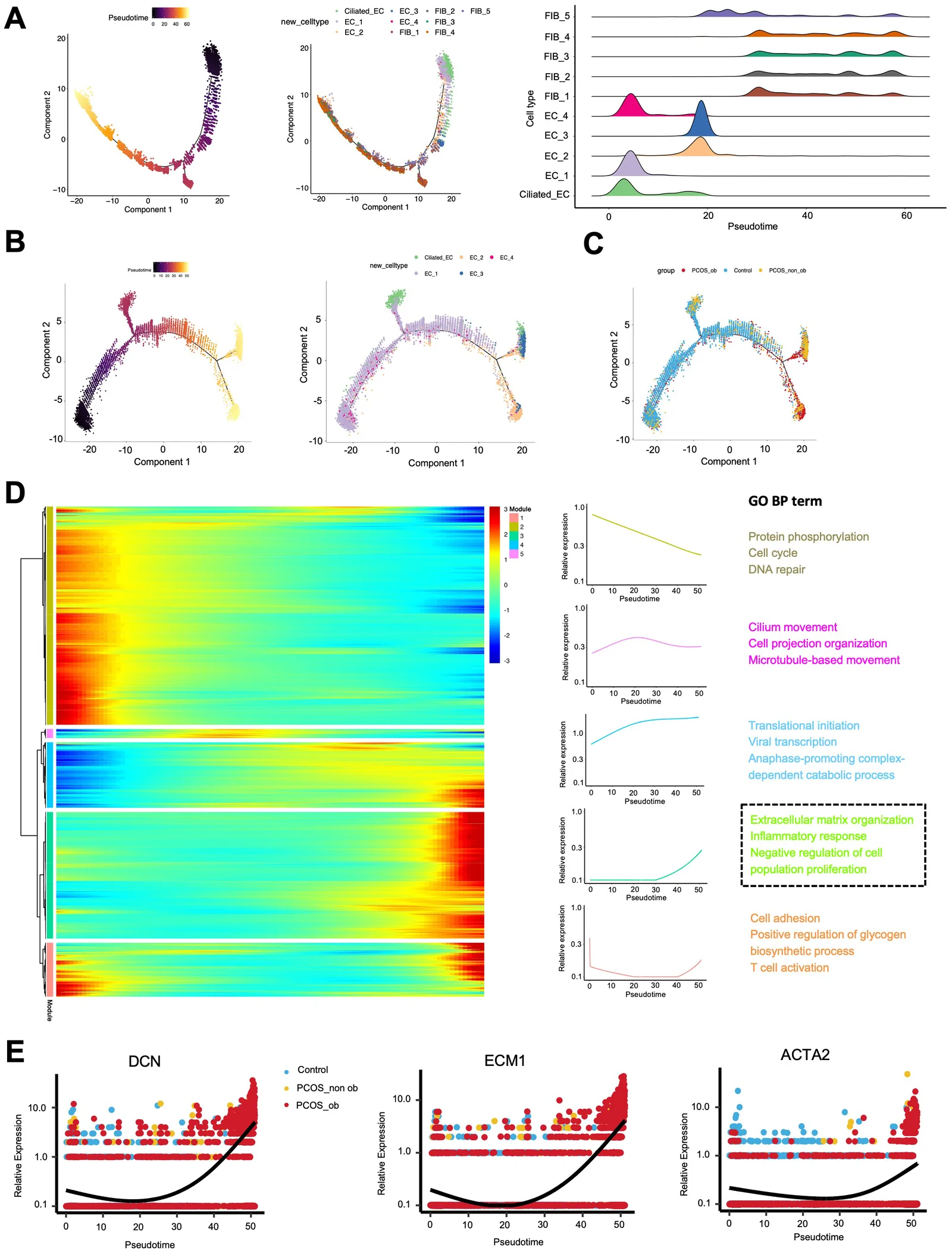
Pseudotime reconstruction of epithelial cells in non-obese PCOS, obese PCOS, and control groups. **(A)** Pseudotime trajectory showing the progression of epithelial cells to fibroblasts. **(B)** Pseudotime trajectory illustrating progression within epithelial cells. **(C)** Pseudotime trajectory showing progression from the control group to the PCOS_non ob and PCOS_ob groups. **(D)** Heatmap displaying highly variable genes in epithelial cells during progression, along with the enriched GO terms for each gene cluster. Dashed boxes highlight the enriched GO terms of upregulated highly variable genes. **(E)** Expression patterns of representative genes (DCN, ECM1, and ACTA2) along the reprogramming trajectory from the control group to the PCOS_non ob and PCOS_ob groups.

Pseudotime reconstruction was performed on ECs to trace their differentiation trajectory during PCOS progression. The trajectory plot reveals a tendency of ECs to differentiate into fibroblast-like cells (**Figure 4A**). Among the epithelial subpopulations, DCN^high^ECM1^high^ EC2 likely originates from the differentiation of EC1 (**Figure 4B**). Using the control group as the baseline, the developmental trajectory from a healthy state to PCOS was mapped (**Figure 4C**). Along this differentiation pathway, downregulated highly variable genes (HVGs) were predominantly associated with protein phosphorylation, cell cycle, and DNA repair processes, while upregulated HVGs were enriched in extracellular matrix (ECM) organization, inflammatory response, and cell proliferation pathways (**Figure 4D**). The expression patterns of ECM-related genes (DCN, ECM1) and the classic fibrosis marker gene ACTA2 followed the developmental progression of PCOS (**Figure 4E**). These results suggest that the differentiation of EC1 into DCN^high^ECM1^high^ EC2 may drive the transition of the endometrium from a healthy to a diseased state, likely explaining the reduced proportion of EC1 and the concurrent increase in DCN^high^ECM1^high^ EC2 in PCOS patients.

### Cell-cell communications between epithelial cells and other subclusters in PCOS patients stratified by obesity

**Figure 5:**
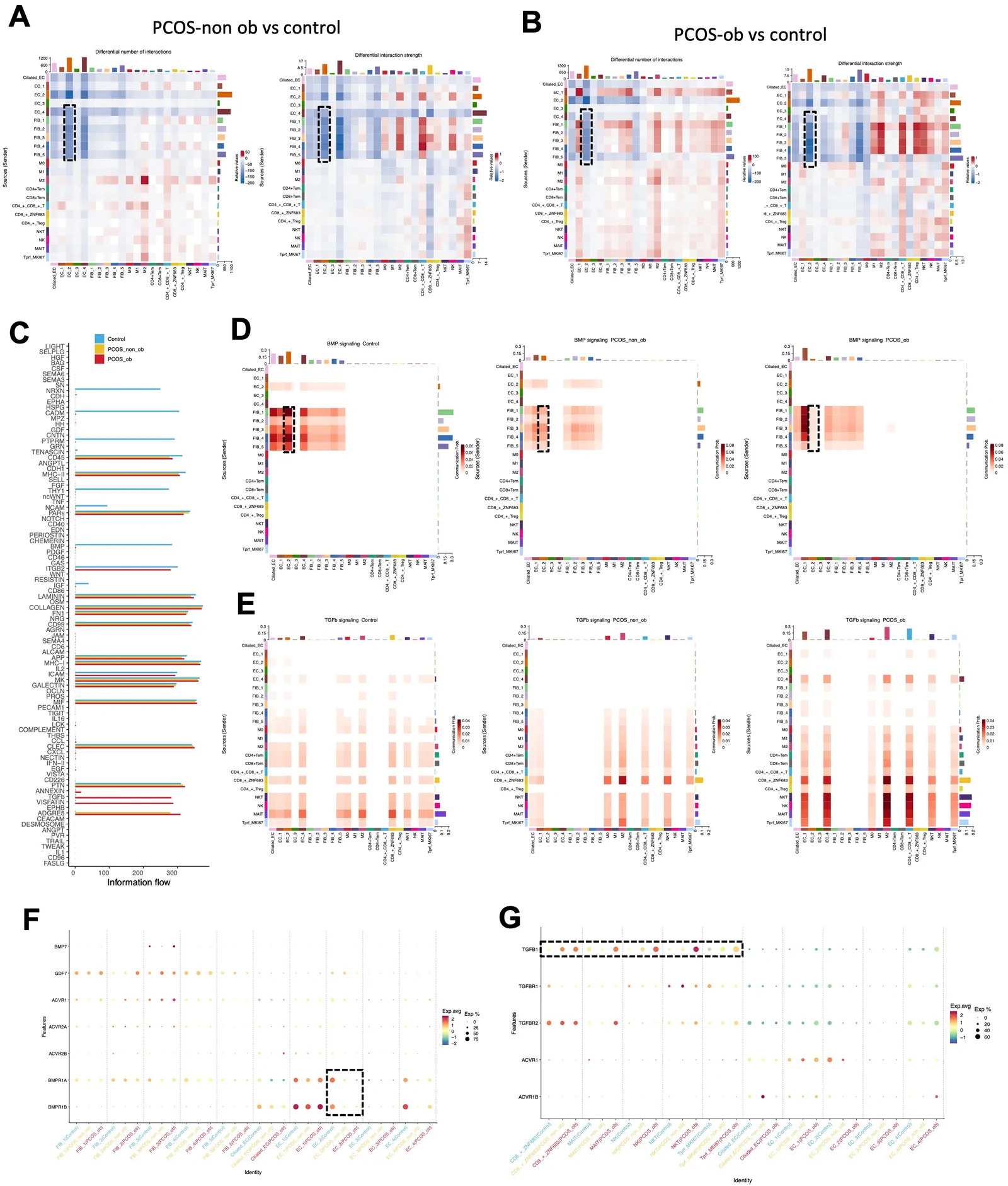
Cell-cell communications between epithelial cells and other cell types in non-obese PCOS, obese PCOS, and control groups. **(A, B)** Heatmap illustrating differential interaction numbers and strengths among all identified subclusters when comparing the PCOS_non ob group or PCOS_ob group with the control group. The colored bars at the top represent the cumulative strength of incoming signals for each subcluster, while those on the right show the cumulative strength of outgoing signals. Bar height indicates the overall difference in interaction numbers and strength between conditions. The color scale indicates whether signal flow in the PCOS group is increased (red) or decreased (blue) relative to the control group. Dashed boxes highlight interactions between FIB subclusters and EC2. **(C)** Information flow of each signaling pathway among the control, PCOS_non ob, and PCOS_ob groups. **(D, E)** Heatmap displaying the strength of BMP and TGFβ signaling pathways across all identified subclusters in the control, PCOS_non ob, and PCOS_ob groups. Dashed boxes highlight interactions between FIB subclusters and EC2. **(F, G)** Dot plots showing the expression of ligand and receptor genes in the BMP and TGFβ signaling pathways across the three groups. Dashed boxes highlight the expression of BMPR1A and BMPR1B in EC2, and TGFβ1 expression in CD8^+^ ZNF683, MAIT, NK, NKT, and Tprt_MKI67 cells.

Given the critical role of intercellular communication in mediating endometrial responses to hormonal imbalance, CellChat analysis was employed to systematically map ligand-receptor-mediated communication networks within and between epithelial, stromal, and immune cell subpopulations. Compared to the control group, the total number of inferred interactions was reduced in both the PCOS_non-ob and PCOS_ob groups (**Supplementary Figure 5A**). Notably, FIB subpopulations showed reduced differential interaction number and strength with DCN^high^ECM1^high^ EC2 in both PCOS groups, whereas their interactions with immune cells, including M2 macrophages and NK cells, were significantly increased (**Figures 5A, B**). The endometrial immune system was more extensively activated in the PCOS_ob group than in the PCOS_non-ob group (**Figure 5B**, **Supplementary Figure 5B**).

**Figure 6:**
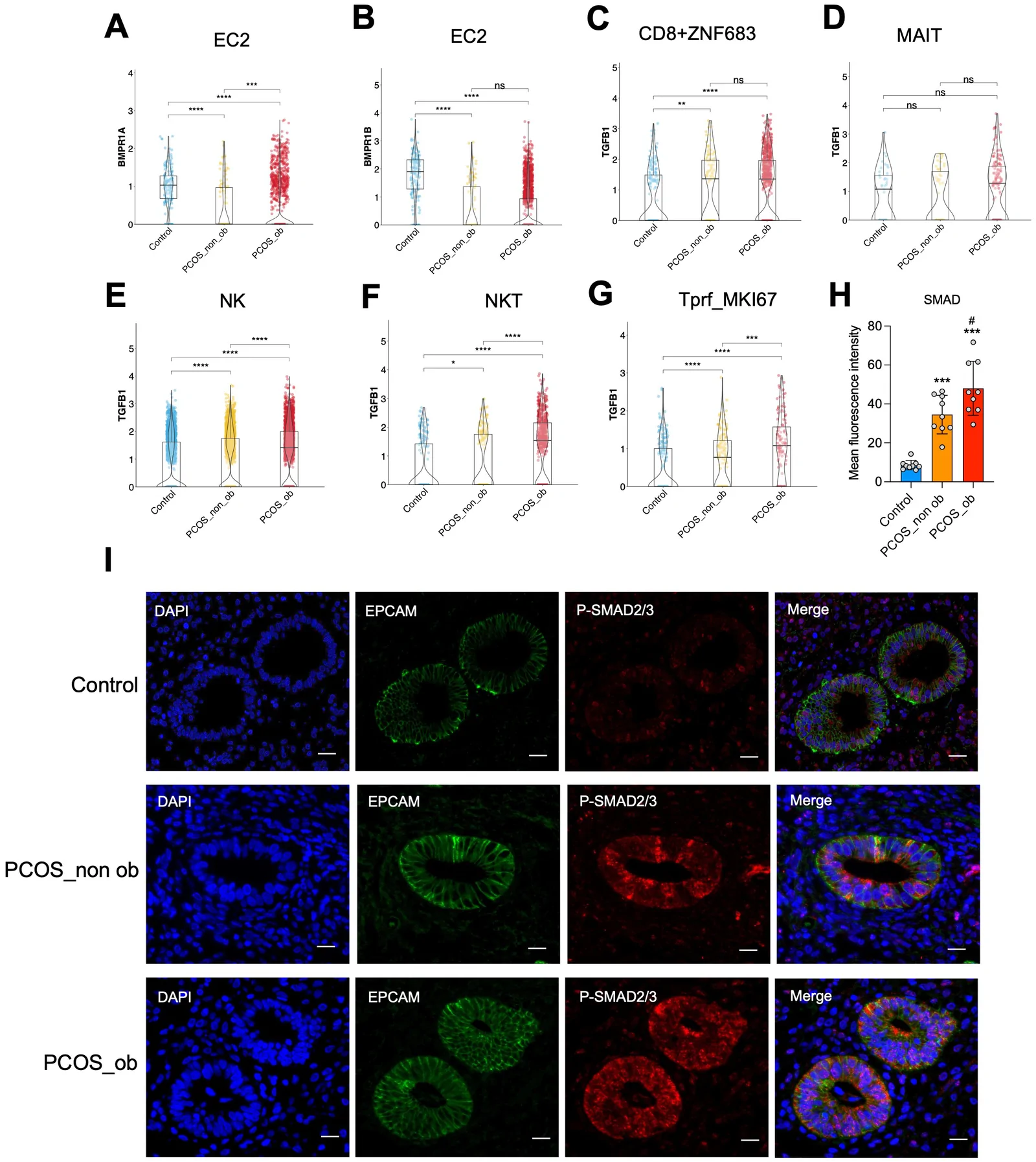
Expression of BMP and TGFβ signaling pathways in non-obese PCOS, obese PCOS, and control groups. **(A, B)** Log-transformed expression of BMPR1A and BMPR1B in DCN^high^ECM1^high^ EC2 cells across the three groups. **(C-G)** Log-transformed expression of TGFβ1 in CD8^+^ ZNF683, MAIT, NK, NKT, and Tprf_MKI67 cells across the three groups. **(H)** The mean fluorescence intensity of P-SMAD2/3 co-expressed with EPCAM in the three groups. ^***^P < 0.001, vs. the control group; ^#^P < 0.05, vs. the PCOS_non ob group. **(I)** Representative immunofluorescence staining for EPCAM **(green)** co-expressed with P-SMAD2/3 (Red). Nuclei were stained with DAPI (blue). Scale bars, 25μm.

A comparison of overall information flow for each signaling pathway in the endometrium among the three groups is shown in **Figure 5C**. Among these pathways, the information flow of the bone morphogenetic protein (BMP) signaling pathway, which regulates cell growth and differentiation, was decreased in both the PCOS_non-ob and PCOS_ob groups. The BMP pathway predominantly occurred between fibroblasts and ECs, and its activity from fibroblasts to DCN^high^ECM1^high^ EC2 was substantially reduced in both PCOS groups (**Figure 5D**, **Supplementary Figure 5C**). The GDF7-(BMPR1A+BMPR2) and GDF7-(BMPR1B+BMPR2) ligand-receptor pairs contributed significantly to endometrial communication (**Supplementary Figure 5E**). Specifically, GDF7, primarily expressed in FIB, did not show substantial changes across the three groups. However, the expression of its receptors, BMPR1A and BMPR1B, significantly decreased in DCN^high^ECM1^high^ EC2 in both the PCOS_non-ob and PCOS_ob groups (**Figure 5F**, **Figures 6A, B**). As a classic fibrosis-related pathway, the information flow of transforming growth factor beta (TGFβ) signaling was increased in the PCOS_ob group (**Figure 5C**). The heatmap reveals that the TGFβ signaling pathway was predominantly expressed in immune cells (**Figure 5E**). The TGFβ1-(TGFβR1+TGFβR2) and TGFβ1-(ACVR1+TGFβR1) ligand-receptor pairs were significantly active in the endometrium (**Supplementary Figure 5F**). Importantly, the expression of the ligand TGFβ1 was significantly upregulated in immune cells, including CD8^+^-ZNF683, NK, NKT, and Tprf-MKI67^+^ cells, in PCOS_non-ob group compared to the control group. The upregulation was even more pronounced in the PCOS_ob group compared to the PCOS_non-ob group (**Figure 5G**, **Figures 6C–G**), indicating that obese phenotype exacerbates the inflammatory and EMT tendencies in PCOS patients. Given that the TGF-β/SMAD2/3 signaling axis is predominantly implicated in promoting fibrosis, we assessed its activation status via immunofluorescence. Notably, p-SMAD2/3 fluorescence intensity was markedly elevated in epithelial cells from both PCOS_non_ob and PCOS_ob groups compared to controls, and intriguingly, the intensity in the PCOS_ob group was significantly higher than that in the PCOS_non_ob group, suggesting a stepwise activation of the TGF-β/SMAD2/3 pathway that correlates with obesity status (**Figures 6H, I**).

### Validation of endometrial gene expression changes in independent cohort and Ishikawa cells

**Figure 7:**
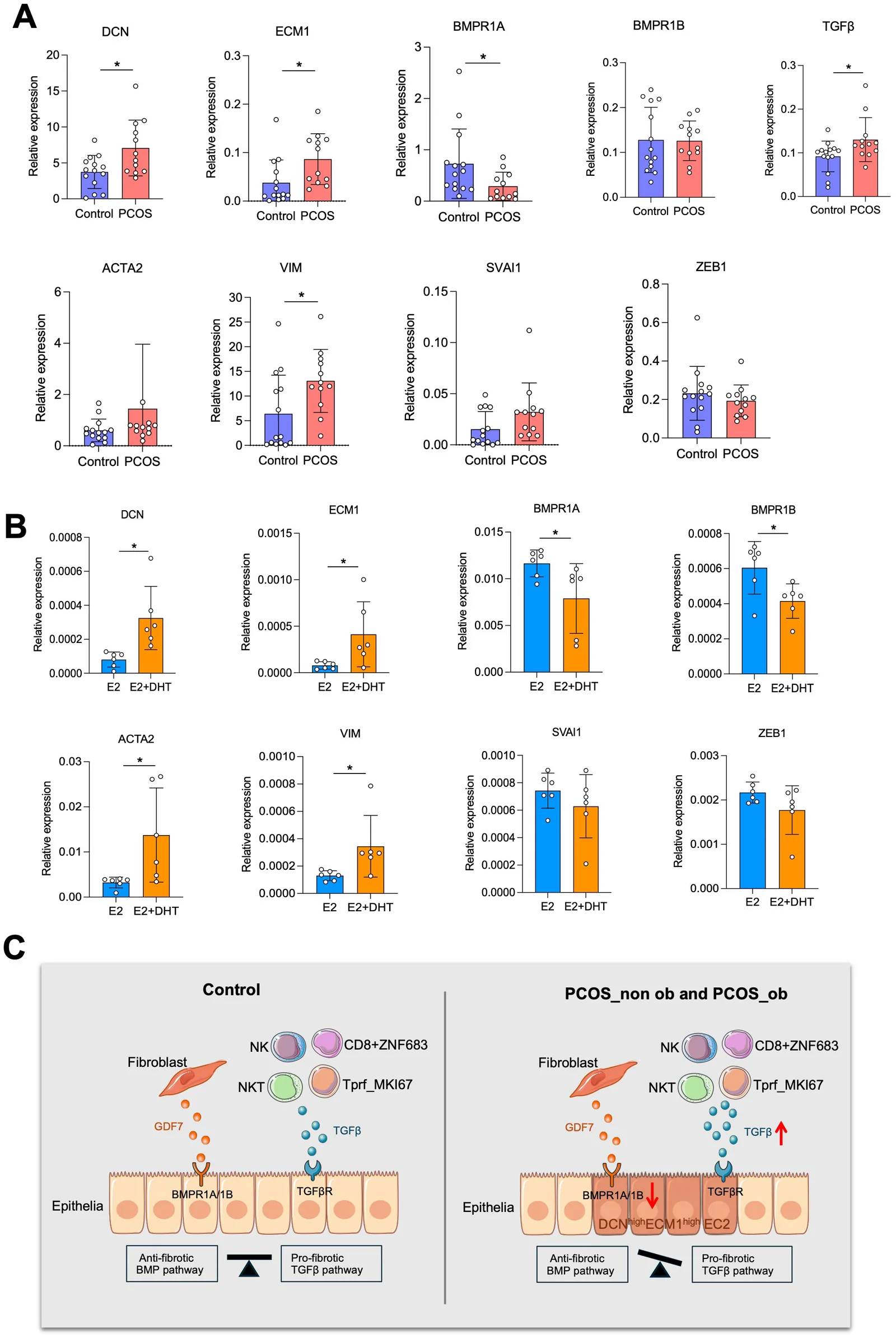
Validation of endometrial gene expression changes in independent cohort and Ishikawa cells. **(A)** qPCR validation using an independent cohort of endometrial samples from PCOS patients (non-obese subgroups, n=12) and matched controls (n=14). **(B)** Relative mRNA expression of genes in Ishikawa cells treated with E2 and DHT. ^*^P < 0.05, vs. the E2-treated group. **(C)** Mechanistic hypothesis diagram: In the control group, the anti-fibrotic BMP signaling pathway and the pro-fibrotic TGF-β signaling pathway maintain a balanced interaction, preserving the structural and functional homeostasis of endometrial epithelial cells. In the PCOS_non ob and PCOS_ob groups, an imbalance arises, marked by decreased BMP receptor expression in epithelial cells and increased TGFβ secretion by immune cells. This imbalance drives epithelial-mesenchymal transition, ultimately resulting in reduced endometrial receptivity.

To further verify the alterations in the expression levels of these genes, we conducted qPCR assays using endometrial samples obtained from an additional independent cohort comprising non-obese PCOS patients (n=12) and matched controls (n=14). Our results demonstrated that the expression of DCN, ECM1, TGFβ1 and VIM was significantly upregulated, while BMPR1A was downregulated in the endometrium of PCOS patients (New **Figure 7A**). Moreover, we treated Ishikawa cells with DHT to induce PCOS-like phenotypes. This treatment significantly upregulated the expression of DCN, ECM1, ACTA2, and VIM, while concurrently downregulating BMPR1A and BMPR1B (**Figure 7B**). Collectively, these results suggest that the imbalance between the anti-fibrotic BMP pathway and the pro-fibrotic TGFβ pathway may drive the fibrotic transformation of normal endometrial ECs in both non-obese and obese PCOS patients (**Figure 7C**).

## Discussion

Endometrial dysfunction significantly contributes to embryo implantation failure in women with PCOS, necessitating an in-depth understanding of its underlying mechanisms and the identification of potential therapeutic targets. This study utilized scRNA-seq to analyze cellular heterogeneity in the proliferative-phase endometrium of healthy controls, non-obese PCOS women, and obese PCOS women. A distinct subpopulation of endometrial ECs exhibiting a tendency for EMT was identified, characterized by high expression of DCN and ECM1 (DCN^high^ECM1^high^ EC2). An increased proportion of these cells may impair EC function, thereby reducing endometrial receptivity in PCOS patients. This pathological process appears to be driven by the activation of pro-fibrotic TGFβ signaling in immune cells and a reduction in anti-fibrotic BMP pathway activity in DCN^high^ECM1^high^ EC2. The findings provide potential therapeutic targets for addressing endometrial dysfunction in PCOS.

In a recent study, Eriksson et al. performed single-nuclei RNA sequencing (snRNA-seq) on frozen proliferative-phase endometrial biopsies from PCOS patients and controls (24). Notably, all participants had a BMI ≥ 25 kg/m^2^, with no significant BMI difference between the PCOS and control groups. In contrast, our study applied scRNA-seq to fresh endometrial biopsies, distinguishing between different BMI profiles: a healthy control group (BMI 18.5–24 kg/m^2^), a non-obese PCOS group (BMI 18.5–24 kg/m^2^), and an obese PCOS group (BMI ≥ 28 kg/m^2^). Eriksson et al. observed an elevated proportion of ECs alongside reduced stromal and lymphoid populations in PCOS endometrial samples. Conversely, our results show a decrease in EC proportion in both non-obese and obese PCOS groups. Despite these differences in cellular composition, consistent with their findings, our study also detected downregulation of ESR1 expression in the endometrial epithelium of PCOS patients, suggesting a conserved molecular alteration regardless of cellular shifts. Both studies highlighted substantial transcriptomic changes in PCOS endometrial ECs, particularly in pathways related to cell adhesion, ECM organization, and integrin-mediated signaling.

DCN, a prominent member of the small leucine-rich proteoglycan (SLRP) family, plays a pivotal role in the ECM, predominantly synthesized by fibroblasts and myoblasts (20). Regarded as an anti-fibrotic factor, DCN neutralizes TGFβ1, a key cytokine driving fibrosis, thereby inhibiting collagen fibrillogenesis and cross-linking to counteract fibrotic progression (20, 21, 25, 26). ECM1, a secreted glycoprotein initially identified in osteogenic stromal cells, is involved in cell differentiation, angiogenesis, and ECM organization (27). Recently, Link et al. highlighted ECM1’s hepatoprotective effect, noting its role in inhibiting latent TGFβ1 activation, suggesting ECM1 or its peptide as potential antifibrotic therapies for chronic liver disease (22). Fan et al. demonstrated in ECM1 knockout mice that ECM1 produced by hepatocytes inhibits TGFβ activation and prevents fibrogenesis in the liver (28). Consequently, DCN and ECM1 are often highly expressed in fibrotic tissues and serves as a biomarker for fibrosis. Intriguingly, our study identified a subpopulation of endometrial ECs characterized by high expression of both DCN and ECM1 (DCN^high^ECM1^high^ EC2). The proportion of DCN^high^ECM1^high^ EC2 cells was elevated in both non-obese and obese PCOS groups, correlating with increased expression of DCN and ECM1. Since DCN and ECM1 are anti-fibrotic genes according to the literature, the observed increase in DCN and ECM1 in the endometrial epithelia does not imply a pathogenic driver role; rather, it most likely reflects (1) a compensatory protective response mounted against overwhelming pro-fibrotic signals (e.g., TGF-β overactivity), or (2) a non-specific consequence of active tissue remodeling, which may elevate their detectable levels without altering their fundamental anti-fibrotic functionality. In contrast, the proportion of glycolipid metabolism-related EC1 cells was reduced, suggesting that the DCN^high^ECM1^high^ EC2 subpopulation may disrupt normal EC function in PCOS patients. Pseudotime analysis further suggested that endometrial ECs in PCOS patients tend to differentiate into a fibroblast-like state, potentially from normal ECs (EC1). ACTA2, encoding α-SMA, a major contractile protein expressed in smooth muscle cells and myofibroblasts (29), correlates with the degree of tissue fibrosis and is frequently used as a biomarker for monitoring fibrotic progression and therapeutic response (30). In the present study, a significant increase in α-SMA protein levels was observed within the endometrial ECs of non-obese and obese PCOS patients, indicating the presence of epithelial fibrosis in PCOS. It should be noted that, owing to the technical difficulties associated with DCN staining, we are unable to definitively conclude that the observed upregulation of α-SMA is specific to the EC2 subset.

Hyperandrogenism, the primary cause of infertility in PCOS, results in follicular developmental disorders due to prolonged exposure to elevated androgen levels, which stimulate chronic low-grade inflammation in the ovaries and activate the NLRP3 inflammasome. This induces follicular dysfunction and ovarian interstitial fibrosis (31, 32). In this study, androgen levels were elevated in non-obese PCOS patients and further increased in obese PCOS patients. KEGG enrichment analysis revealed significant activation of apoptosis- and inflammation-related pathways in DCN^high^ECM1^high^ EC2 cells. These findings suggest that endometrial epithelial fibrosis in PCOS may also be driven by a hyperandrogenic state. Ye et al. confirmed the presence of fibrosis in the proliferative-phase endometrium of both PCOS patients and PCOS-like mouse models (33). Moreover, they demonstrated that GPX4 deficiency-induced ferroptosis promotes ECM remodeling and excessive collagen deposition by activating the TGFβ1/Smad2/3 signaling pathway, thereby accelerating fibrotic progression. In addition, the liver, adipose tissue, and ovaries interact with each other through autophagy dysregulation, chronic low-grade inflammation, and metabolic disturbances, thereby inducing insulin resistance and hyperandrogenemia, which in turn aggravate the PCOS phenotype (34).

TGFβ plays a pivotal role in maintaining homeostasis across various tissues, regulating processes such as inflammation, cell proliferation, differentiation, and wound healing (35, 36). Numerous studies have demonstrated that TGFβ promotes fibroblast activation and proliferation, resulting in ECM deposition (37, 38). Elevated TGFβ expression is frequently associated with the development and severity of fibrotic diseases (39–41). BMPs, initially identified for their ability to induce bone formation (42, 43), interact with TGFβ signaling in a dynamic, bidirectional manner, exhibiting both synergistic and antagonistic effects to co-regulate fibrosis-promoting processes, such as EMT (44, 45). Chen et al. showed that exogenous recombinant BMP7 alleviates histopathological features of fibrotic lesions in a schistosomiasis-induced hepatic fibrosis model, with this effect closely linked to the downregulation of TGFβ1, p-SMAD2, and the pro-fibrotic marker α-SMA (46). Monsivais et al. demonstrated using BMP receptor knockout mice that uterine BMP signaling, through an ACVR2A-SMAD1/SMAD5 axis, is crucial for endometrial receptivity and implantation (47). In endometriosis, impaired BMP signaling directly disrupts the endometrial decidualization process, contributing to reduced fertility (48). In the present study, significantly elevated TGFβ1 expression was observed in endometrial immune cells, including CD8^+^ ZNF683, NK, NKT, and Tprf_MKI67, in both non-obese and obese PCOS patients. Moreover, p-SMAD2/3 was markedly activated in the epithelial cells of PCOS groups. On the contrast, expression of BMP receptors BMPR1A and BMPR1B was reduced in the DCN^high^ECM1^high^ EC2 subpopulation. These findings suggest an inflammatory state in the endometrium of PCOS patients, characterized by an imbalance in fibrosis-related mediators—namely, elevated pro-fibrotic TGFβ1 and diminished anti-fibrotic BMP signaling—potentially driving a pro-fibrotic shift in endometrial ECs.

This study has several limitations. Firstly, the sample size for scRNA-seq is relatively small, which may reduce the power to detect subtle heterogeneity, particularly when stratifying by obesity status. Therefore, future validation in larger, independent cohorts using targeted methods is essential to confirm our key findings. Secondly, the grouping criteria did not include a transitional overweight group (BMI 24-27.9 kg/m²), which may introduce confounding due to the continuous effect of the BMI gradient. Although our comparison between lean PCOS patients and healthy normal-weight controls suggests that the endometrial pathological changes are attributable to PCOS, the absence of a simple obesity group precludes us from determining the isolated effect of obesity alone, as well as from directly comparing simple obesity with obese PCOS to assess potential additive effects of PCOS. Future studies incorporating a full spectrum of BMI categories and metabolic phenotypes are warranted to confirm causality. Lastly, future prospective studies should enroll PCOS patients before and after effective interventions (e.g., lifestyle modification or metformin), and examine whether the candidate molecular markers or signaling pathways identified in this study are reversed in parallel with symptom improvement.

In conclusion, this study identifies, via scRNA-seq, a distinct subpopulation of EMT-prone ECs (DCN^high^ECM1^high^ EC2) in the endometrium. The increased presence of these cells in non-obese and obese PCOS patients suggests a cellular basis for impaired receptivity and subsequent implantation failure. Notably, obese phenotype further exacerbates these endometrial alterations in PCOS patients. An imbalance between elevated TGFβ and diminished BMP pathway activities as a likely driver of this pro-fibrotic shift. Understanding the mechanisms underlying this TGFβ-BMP dysregulation and targeting them therapeutically could pave the way for novel treatments addressing endometrial dysfunction in PCOS.

## Data Availability

The datasets presented in this study can be found in online repositories. The names of the repository/repositories and accession number(s) can be found in the article/**Supplementary Material**.
