# Supplementary material for: Single-cell RNA sequencing reveals a pro-fibrotic epithelial subpopulation contributing to endometrial dysfunction in polycystic ovary syndrome
Source: Front Endocrinol (Lausanne). 2026 Sep 3;17:1873463. doi: 10.3389/fendo.2026.1873463 (PMC13581741; doi:10.3389/fendo.2026.1873463)
Supplement: Supplementary file 1 [file SupplementaryFile1.docx]

**Supplementary Data**

**Table S1. Baseline characteristics of participants for sc-RNA seq.**

|  | Control group | PCOS_non ob group | PCOS_ob group |
| --- | --- | --- | --- |
| Age (year) | 31.67±1.53 | 30.0±2.0 | 29.33±4.73 |
| BMI (kg/m^2^) | 21.88±1.26 | 21.45±1.89 | 29.3±2.0^***##^ |
| LH (IU/L) | 3.70±0.88 | 6.12±1.75^P=0.09^ | 9.28±1.99^*^ |
| FSH (IU/L) | 7.65±1.25 | 5.10±2.23 | 6.03±1.37 |
| Testosterone (ng/ml) | 0.23±0.04 | 0.39±0.09^P=0.05^ | 0.65±0.25^*^ |
| Estradiol (pg/ml) | 36.99±9.15 | 39.87±4.62 | 48.42±4.77 |
| AMH (ng/ml) | 3.97±1.05 | 10.72±1.76^**^ | 6.79±2.09 |
| Insulin (uIU/mL) | 4.04±0.37 | 12.30±1.91^**^ | 12.39±5.15^*^ |
| Fasting glucose (mmol/L) | 4.57±0.42 | 4.40±0.26 | 5.27±0.32^#^ |
| HOMA-IR | 0.82±0.09 | 2.41±0.46^**^ | 2.86±1.01^*^ |
| Menstrual cycle (day) | 28-30 | 60-120 | 60-180 |
| Clinical hyperandrogenism | No | No | No |
| Medication use | No | No | No |
| Polycystic ovarian morphology | No | ≥ 20 antral follicles per ovary | ≥ 20 antral follicles per ovary |

Note: P=0.09, P=0.05, ^*^P<0.05, ^**^P<0.01, ^***^P<0.001, vs. control group. ^#^P<0.05, ^##^P<0.01, vs. PCOS_non ob group. n=3 per group.

**Table S2. The number of cells per sub-cluster per group.**

| Cell number | Ciliated EC | EC1 | EC2 | EC3 | EC4 |
| --- | --- | --- | --- | --- | --- |
| Control | 275 | 5095 | 167 | 32 | 231 |
| PCOS_non ob | 137 | 156 | 105 | 185 | 3 |
| PCOS_ob | 51 | 1205 | 2425 | 546 | 43 |

**Figure S1.** Feature plots illustrating the marker gene expression characterizing each major cluster of endometrial cells.

**Figure S2.** Distribution of major clusters visualized by UMAP in individual endometrial samples from the control, PCOS_non ob, and PCOS_ob groups.

**Figure S3.** (A) UMAP showing the subcluster distribution of epithelial cells. (B) Log-transformed expression of DCN, ECM1, and CXCL2 characterizing DCN^high^ECM1^high^ EC2. (C) GSVA of epithelial subclusters. (D) UMAP showing the subcluster distribution of epithelial cells in the control, PCOS_non ob, and PCOS_ob groups. (E) Bar plots depicting the proportion of epithelial subclusters in individual endometrial samples from the control, PCOS_non ob, and PCOS_ob groups.

**Figure S4.** (A) GSVA of fibroblast (FIB) subclusters. (B) Violin plots showing the log-transformed expression of ESR1, PGR, and AR in epithelial subclusters from the control, PCOS_non ob, and PCOS_ob groups. *P < 0.05, **P < 0.01, ***P < 0.001, *vs.* control group.

**Figure S5.** (A) The number and strength of inferred interactions in endometrial cells from the control, PCOS_non ob, and PCOS_ob groups. (B) Heatmap showing differential interaction strength among all identified subclusters when comparing the PCOS_ob group and the PCOS_non ob group. The colored bars at the top represent the cumulative strength of incoming signals for each subcluster, while those on the right depict the cumulative strength of outgoing signals. Bar height reflects the overall difference in interaction number and strength between conditions. The color scale indicates whether signal flow in the PCOS_ob group is increased (red) or decreased (blue) relative to the control. (C, D) The strength of ligand-receptor interactions of BMP and TGFβ signaling pathways in specific subclusters from the control, PCOS_non ob, and PCOS_ob groups. (E, F) Contribution of each ligand-receptor pair of BMP and TGFβ signaling pathways in the control, PCOS_non ob, and PCOS_ob groups.
